# Regulating the Way to Obesity: Unintended Consequences of Limiting Sugary Drink Sizes

**DOI:** 10.1371/journal.pone.0061081

**Published:** 2013-04-10

**Authors:** Brent M. Wilson, Stephanie Stolarz-Fantino, Edmund Fantino

**Affiliations:** University of California San Diego, La Jolla, California, United States of America; University of Missouri-Kansas City, United States of America

## Abstract

**Objectives:**

We examined whether a sugary drink limit would still be effective if larger-sized drinks were converted into bundles of smaller-sized drinks.

**Methods:**

In a behavioral simulation, participants were offered varying food and drink menus. One menu offered 16 oz, 24 oz, or 32 oz drinks for sale. A second menu offered 16 oz drinks, a bundle of two 12 oz drinks, or a bundle of two 16 oz drinks. A third menu offered only 16 oz drinks for sale. The method involved repeated elicitation of choices, and the instructions did not mention a limit on drink size.

**Results:**

Participants bought significantly more ounces of soda with bundles than with varying-sized drinks. Total business revenue was also higher when bundles rather than only small-sized drinks were sold.

**Conclusions:**

Our research suggests that businesses have a strong incentive to offer bundles of soda when drink size is limited. Restricting larger-sized drinks may have the unintended consequence of increasing soda consumption rather than decreasing it.

## Introduction

Both waistlines and portion sizes have increased substantially since the 1970s [1], [2], [3], [4], [5]. Larger servings provide more calories, and previous research has shown that increased portion size leads to greater consumption [6], [7]. Numerous studies have also focused on the association between soft drink consumption and increased energy intake without nutrition [8]. In an effort to reduce the consumption of sugary beverages and to combat the rising obesity epidemic, New York City passed a measure restricting the sale of sugary drinks larger than 16 ounces [9]. The restriction should be effective if it results in smaller portion sizes being available and lowers consumption norms [10].

The restriction, however, would not prevent larger-sized drinks from being sold as bundles of smaller-sized drinks. A large, 32 ounce drink could be replaced with a bundle of two, 16 ounce drinks. Prior research has shown that package size only influences use when different sizes have different unit costs [11]. If the price per ounce of soda does not change between the varying conditions, soda consumption may not decline when bundles are offered in lieu of larger cup sizes. Previous work has shown that mandates intended to improve eating habits may have the unintended consequence of increasing consumption [12], [13].

## Method

### Participants

One hundred (24 male, 76 female) undergraduate students at the University of California, San Diego participated in this experiment for course credit. Participants had an average age of 20.78 and ranged from 18 to 39 years old. All participants provided written informed consent. The study was conducted according to the Institutional Review Board of the University of California, San Diego guidelines for the protection of human participants.

### Materials

Different menu choices were presented on paper. Line drawings of cups, containers of popcorn, and slices of pizza were presented along with the name of the item and the price of each item or bundle. Items could be purchased at a fast food restaurant, movie theater, or stadium. Each food or beverage option had a quantity line for subjects to indicate how many of each item or bundle they would like to purchase. Each participant saw eight or nine different choice menus, and the order of these menus was randomized. Three of the choice menus were critical trials. These three critical trials all had fast food restaurant as the location. The Unregulated menu presented a 16 oz soda for $1.59, a 24 oz soda for $1.79, and a 32 oz soda for $1.99. (These prices were obtained from McDonald's menus at the time of the study.) The Bundle menu presented a 16 oz soda for $1.59, two 12 oz sodas for $1.79, and two 16 oz sodas for $1.99. The No Bundle menu presented only a 16 oz soda for $1.59.

### Procedure

In a behavioral simulation, participants were offered varying food and drink menus. The method involved repeated elicitation of choices. Participants were given a specified location (fast food restaurant, movie theater, or stadium) and asked to select the item or items they would purchase for themselves. They were instructed to write a number on the quantity line below each item indicating how many they would purchase. They were told to write at least one number for each menu and to write the number zero on the first quantity line if they did not believe they would purchase anything from that menu. The instructions did not mention a restriction on large drinks nor were participants asked how they would react to a restriction, which could bias responses.

## Results

**Figure 1:**
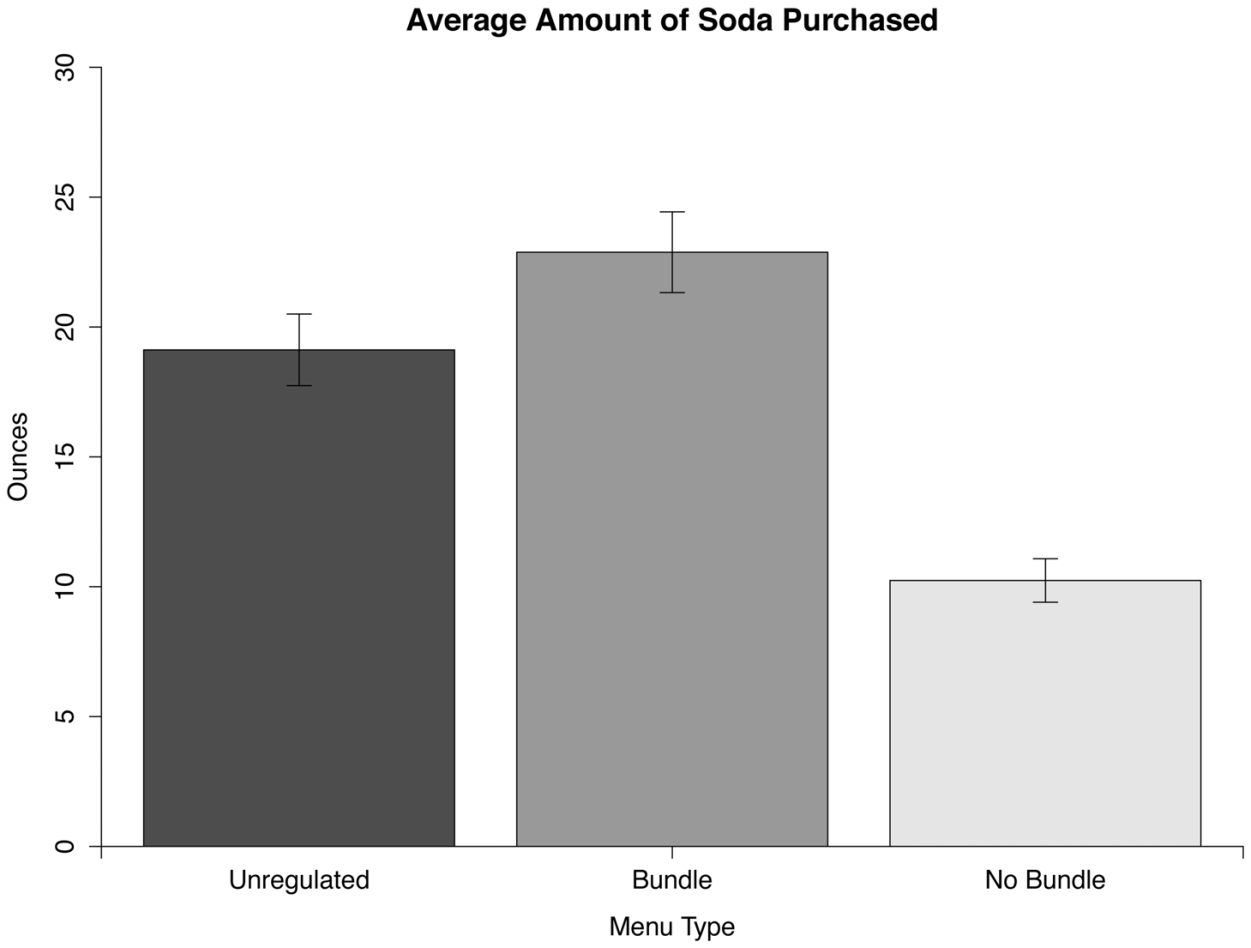
Average amount of soda purchased by each participant in the three conditions. Error bars represent standard error.

Participants bought significantly more ounces of soda from the Bundle menu than from the Unregulated menu according to a 2-tailed paired *t* test, *t*(99) = 4.03, *p*<0.001, Cohen's *d* = 0.40. Participants, however, bought significantly fewer ounces of soda from the No Bundle menu than from the Unregulated menu according to a 2-tailed paired *t* test, *t*(99) = 7.94, *p*<0.001, *d* = 0.79. Figure 1 shows the amount of soda purchased in the three conditions.

**Figure 2:**
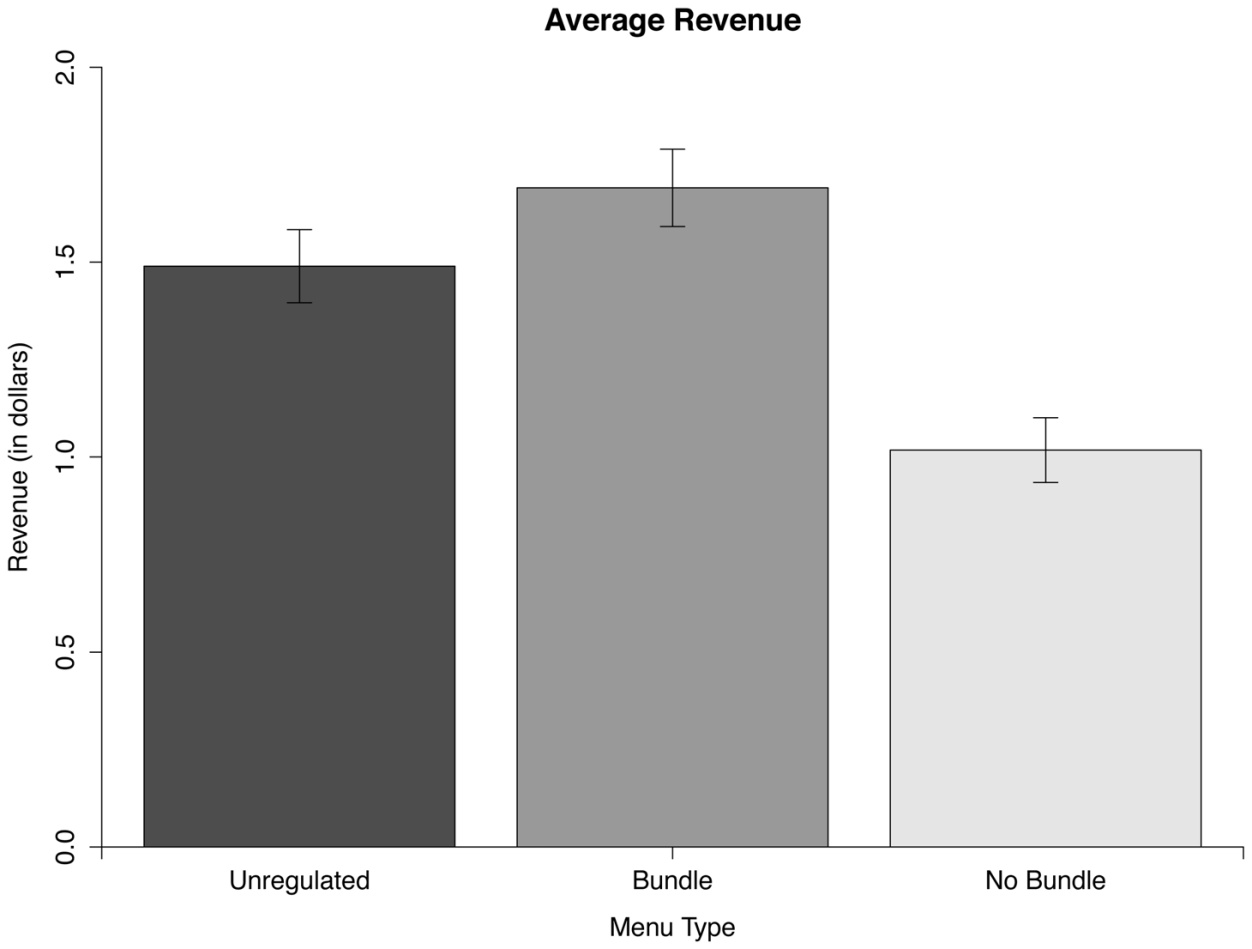
Average revenue from each participant in the three conditions. Error bars represent standard error.

Revenue was significantly higher for the Bundle menu than for the Unregulated menu according to a 2-tailed paired *t* test, *t*(99) = 3.48, *p* = 0.001, *d* = 0.35. Revenue was also significantly higher for the Bundle menu than for the No Bundle menu according to a 2-tailed paired *t* test, *t*(99) = 7.24, *p*<0.001, *d* = 0.72. Figure 2 shows the revenue for the three conditions. All *p*-values were Bonferroni corrected.

This pattern of results was the same for both male and female participants. Females bought significantly more ounces of soda from the Bundle menu than from the Unregulated menu, *t*(75) = 2.94, *p* = 0.009. Females also bought significantly fewer ounces of soda from the No Bundle menu than from the Unregulated menu, *t*(75) = 6.66, *p*<0.001. Similarly, males bought significantly more ounces of soda from the Bundle menu than from the Unregulated menu, *t*(23) = 2.87, *p* = 0.017. Additionally, males bought significantly fewer ounces of soda from the No Bundle menu than from the Unregulated menu, *t*(23) = 4.41, *p*<0.001.

Revenue from females was significantly higher for the Bundle menu than for the Unregulated menu, *t*(75) = 2.60, *p* = 0.022. Revenue from females was significantly higher for the Bundle menu than for the No Bundle menu, *t*(75) = 5.57, *p*<0.001. Similarly, revenue from males was significantly higher for the Bundle menu than for the Unregulated menu, *t*(23) = 2.40, *p* = 0.0496. Revenue from males was also significantly higher for the Bundle menu than for the No Bundle menu, *t*(23) = 4.97, *p*<0.001. All *p*-values were Bonferroni corrected.

Twenty-one percent of participants chose to buy “zero sodas” in the Unregulated condition. Sixteen percent of participants chose to buy “zero sodas” in the Bundle condition. Thirty-eight percent of participants chose to buy “zero sodas” in the No Bundle condition. This is consistent with the other results wherein the No Bundle menu is associated with decreased consumption and the Bundle menu is associated with increased consumption.

## Discussion

These data suggest that a sugary drink restriction may not be effective in reducing consumption when businesses are able to sell bundles of soda that add up to the original, larger drink sizes. Proponents of the New York City sugary drink limit are likely anticipating only the small 16 oz size being offered with the medium and large sizes being eliminated from the menu. They may, therefore, be concerned if businesses convert their jumbo-sized sugary drinks into multiple, smaller packages of sugary drinks. In our experiment, businesses had an average revenue of $1.69 when they offered drink bundles but an average revenue of $1.02 when only 16 oz drink sizes were offered. These results show that businesses should earn significantly more revenue when bundles are offered than when small drink sizes alone are offered. This means restaurants have a strong incentive to convert their original-sized drinks into bundles so they do not lose a major source of revenue.

Revenue actually increased when bundles were offered as compared to sales when varying-sized drinks were offered. This suggests that businesses could readily recover any additional costs associated with supplying bundles rather than varying-sized drinks. For example, the extra cup and lid for a bundle would cost only 3.6 cents more to buy from Costco than the individual larger cup. This cost would be even smaller for larger businesses. The quantity of soda purchased has been shown to be influenced by price changes [14], [15], and prices would still need to be competitive with convenience stores not subject to the drink limit. Prices, therefore, would not necessarily be expected to rise when businesses begin offering bundles.

One limitation of the present study is that actual consumption was not measured. This study also did not differentiate between diet soda and regular soda because we did not want this distinction to bias responding. Even though study participants were instructed that the food and drink purchases were for their own consumption, people may have an incentive to share drinks when bundles are offered. As the policy takes effect, there may be other factors that influence drink purchasing (such as the inconvenience of carrying multiple cups). However, this research shows a potential unintended consequence that may need to be considered in future policy making.

Even with the restriction, New York State Senator Daniel Squadron has said that “those who want to drink more will still be able to go ahead and have two” [16]. Our study shows that when larger drink sizes are offered as bundles, people are very likely to go ahead and do just that.
